# Teledentistry Implementation During the COVID-19 Pandemic: Scoping Review

**DOI:** 10.2196/39955

**Published:** 2022-07-21

**Authors:** Man Hung, Martin S Lipsky, Teerarat N Phuatrakoon, Mindy Nguyen, Frank W Licari, Elizabeth J Unni

**Affiliations:** 1 College of Dental Medicine Roseman University of Health Sciences South Jordan, UT United States; 2 College of Dental Medicine Roseman University of Health Sciences Henderson, NV United States; 3 Division of Public Health University of Utah Salt Lake City, UT United States; 4 School of Business University of Utah Salt Lake City, UT United States; 5 College of Social Work University of Utah Salt Lake City, UT United States; 6 Department of Biology University of Utah Salt Lake City, UT United States; 7 Department of Educational Psychology University of Utah Salt Lake City, UT United States; 8 Department of Orthopaedic Surgery Operations University of Utah Salt Lake City, UT United States; 9 Department of Veterans Affairs Medical Center Veterans Affairs Salt Lake City Health Care System Salt Lake City, UT United States; 10 Huntsman Cancer Institute Salt Lake City, UT United States; 11 Institute on Aging Portland State University Portland, OR United States; 12 College of Pharmacy Touro University New York City, NY United States

**Keywords:** teledentistry, telehealth, COVID-19, pandemic, innovation, implementation, dental profession

## Abstract

**Background:**

COVID-19 spreads via aerosol droplets. The dental profession is at high risk of contracting the virus since their work includes treatment procedures that produce aerosols. Teledentistry offers an opportunity to mitigate the risk to dental personnel by allowing dentists to provide care without direct patient contact.

**Objective:**

The purpose of this scoping review was to examine the implementation, challenges, strategies, and innovations related to teledentistry during the COVID-19 pandemic lockdown.

**Methods:**

This scoping review evaluated teledentistry use during the pandemic by searching for articles in PubMed and Google Scholar using the search terms teledentistry, tele-dentistry, covid-19, coronavirus, telehealth, telemedicine, and dentistry. Inclusion criteria consisted of articles published in English from March 1, 2020, to April 1, 2022, that were relevant to dentistry and its specialties, and that included some discussion of teledentistry and COVID-19. Specifically, the review sought to explore teledentistry implementation, challenges, strategies to overcome challenges, and innovative ideas that emerged during the pandemic. It followed the 2020 Preferred Reporting Items for Systematic reviews and Meta-Analyses for Scoping Reviews (PRISMA-ScR). This approach is organized into 5 distinct steps: formulating a defined question, using the question to develop inclusion criteria to identify relevant studies, an approach to appraise the studies, summarizing the evidence using an explicit methodology, and interpreting the findings of the review.

**Results:**

A total of 32 articles was included in this scoping review and summarized by article type, methodology and population, and key points about the aims; 9 articles were narrative review articles, 10 were opinion pieces, 4 were descriptive studies, 3 were surveys, 2 were integrative literature reviews, and there was 1 each of the following: observational study, systematic review, case report, and practice brief. Teledentistry was used both synchronously and asynchronously for virtual consultations, often employing commercial applications such as WhatsApp, Skype, and Zoom. Dental professionals most commonly used teledentistry for triage, to reduce in-person visits, and for scheduling and providing consultations remotely. Identified challenges included patient and clinician acceptance of teledentistry, having adequate infrastructure, reimbursement, and security concerns. Strategies to address these concerns included clinician and patient training and utilizing Health Insurance Portability and Accountability Act-compliant applications. Benefits from teledentistry included providing care for patients during the pandemic and extending care to areas lacking access to dental care.

**Conclusions:**

Pandemic lockdowns led to new teledentistry implementations, most commonly for triage but also for follow-up and nonprocedural care. Teledentistry reduced in-person visits and improved access to remote areas. Challenges such as technology infrastructure, provider skill level, billing issues, and privacy concerns remain.

## Introduction

COVID-19 started in Wuhan, China in December 2019 and quickly spread worldwide [1]. Though genetically similar to SARS coronavirus 2, COVID-19 developed different characteristics, namely rapid upper respiratory tract replication and asymptomatic transmission [2]. Transmission occurs by droplets spread from infected individuals coughing and sneezing [3,4]. Individuals then contract the infection from droplet inhalation or by direct contact of the virus with mucous membranes (eg, oral cavity, nose, and eye) [1,3,4]. Many countries employed lockdowns to mitigate viral spread, including the United States in March 2020 [5].

Transmission through respiratory droplets is a concern for dental professionals and their staff since dental procedures can produce aerosols, which increase the risk of spreading COVID-19 [6]. During the pandemic lockdown, it was important for dental clinics to remain operational to meet community needs. Most dental clinics provided urgent care but deferred elective procedures to reduce disease spread. One option, teledentistry, or the provision of dental care through distance technology, became a significant tool for triaging patients and providing dental care in a safe environment [7].

Telemedicine is defined as the practice of using videoconferencing technologies to diagnose and provide advice about treatment over a distance [8]. In the late 1980s, teledentistry was first introduced as a subcategory of telemedicine [8]. Since its introduction, improved technology has made it possible for more dental patients to be managed without an in-person visit. Jampani et al [8] recognized teledentistry as a way to increase consultation capabilities through the sharing of photos, radiographs, and clinical information; improving communication between dental professionals; and extending care for patients living in rural areas where specialists may not be readily accessible. Prepandemic, teledentistry emerged as a method for triaging patients and providing long-distance care [7]. However, compared with other health care disciplines, dentistry has been slower to utilize telemedicine and adopt teledentistry as a mainstream tool [9].

The COVID-19 pandemic and lockdowns generated unparalleled economic and social disruption, creating an opportunity for expanding telemedicine [10]. In oral health, teledentistry became one strategy to mitigate the pandemic’s impact by reducing face-to-face visits while supplementing patient care [11]. The wider use of teledentistry during the pandemic makes it important to understand issues related to its enhanced use.

The purpose of this scoping review was to evaluate teledentistry use during the pandemic. Specifically, the review sought to answer the following 4 questions:

1. How was teledentistry implemented during the COVID-19 pandemic?
2. What challenges occurred when implementing teledentistry during the COVID-19 pandemic?
3. What strategies were used to overcome these challenges?
4. Were there innovative ideas resulting from the implementation of teledentistry?

## Methods

Scoping reviews map the available evidence of a content area. They are useful for identifying knowledge gaps, generating new research questions, guiding practice, and informing policy makers about an emerging area [12]. This scoping review explored the implementation and expansion of teledentistry during the COVID-19 pandemic.

The review followed the 2020 Preferred Reporting Items for Systematic reviews and Meta-Analyses for Scoping Review (PRISMA-ScR; Multimedia Appendix 1) [13]. This approach is organized into 5 distinct elements or steps: formulating a defined question, using the question to develop inclusion criteria to identify relevant studies, an approach to appraise the studies, summarizing the evidence using an explicit methodology, and interpreting the findings of the review.

To identify relevant articles, 2 authors (MN and TNP) systematically searched PubMed and Google Scholar to identify potentially relevant literature. Google Scholar was included since the growing literature supports the value of incorporating Google Scholar in relationship to other indexing databases such as PubMed [14,15]. For the initial step, we used the following article search terms: ((teledentistry) OR (tele-dentistry) OR (telehealth) OR (telemedicine)) AND ((covid-19[MeSH Terms]) OR (coronavirus)) AND (dentistry)) AND ((“2020/03/01”[Date - Publication]: “2022/04/01”[Date - Publication])). To broaden the search and capture other articles meeting the inclusion criteria, 2 authors (MN and TNP) reviewed the bibliographies of articles obtained from the initial search for additional papers that might be relevant to the review.

Inclusion criteria consisted of (1) articles published from March 2020 (the start of the pandemic lockdown in the United States) to April 2022 that evaluated the implementation or the discussion of teledentistry, (2) articles published in English, (3) articles relevant to dentistry or its specialties, (4) publications in a peer-reviewed journal, and (5) content related to the COVID-19 pandemic. Articles with little or no mention of teledentistry or articles that did not link to any of the research questions (ie, the implementation of teledentistry, challenges with implementation, strategies to address challenges, and innovative models of teledentistry) were excluded. Earlier teledentistry reviews noted a paucity of published research and that a substantial portion of publications was descriptive [9]. Therefore, to capture all the peer-reviewed teledentistry literature that addressed the study aims, this review included descriptive studies and review articles in addition to hypothesis-driven research. To explore the views of teledentistry thought leaders, the search also included opinion pieces such as essays, editorials, and letters to the editor.

After obtaining abstracts for the identified articles, 2 authors (MN and TNP) reviewed them for possible inclusion. Full-text articles of these abstracts were retrieved and reviewed by 2 authors (MN and TNP) to determine if an article met the inclusion criteria. If the authors MN and TNP differed about including an article, they discussed and resolved these differences. If authors encountered difficulties with classifying specific articles or research methodology, authors MH and MSL were consulted for guidance. After all the articles were identified, authors MN and TNP separately read each article to identify key points that addressed the study questions. After identifying key points, authors MN, TNP, and MSL reviewed the key points and resolved any differences. MH reviewed the final table for consistency and quality.

## Results

### Search Strategy

**Figure 1:**
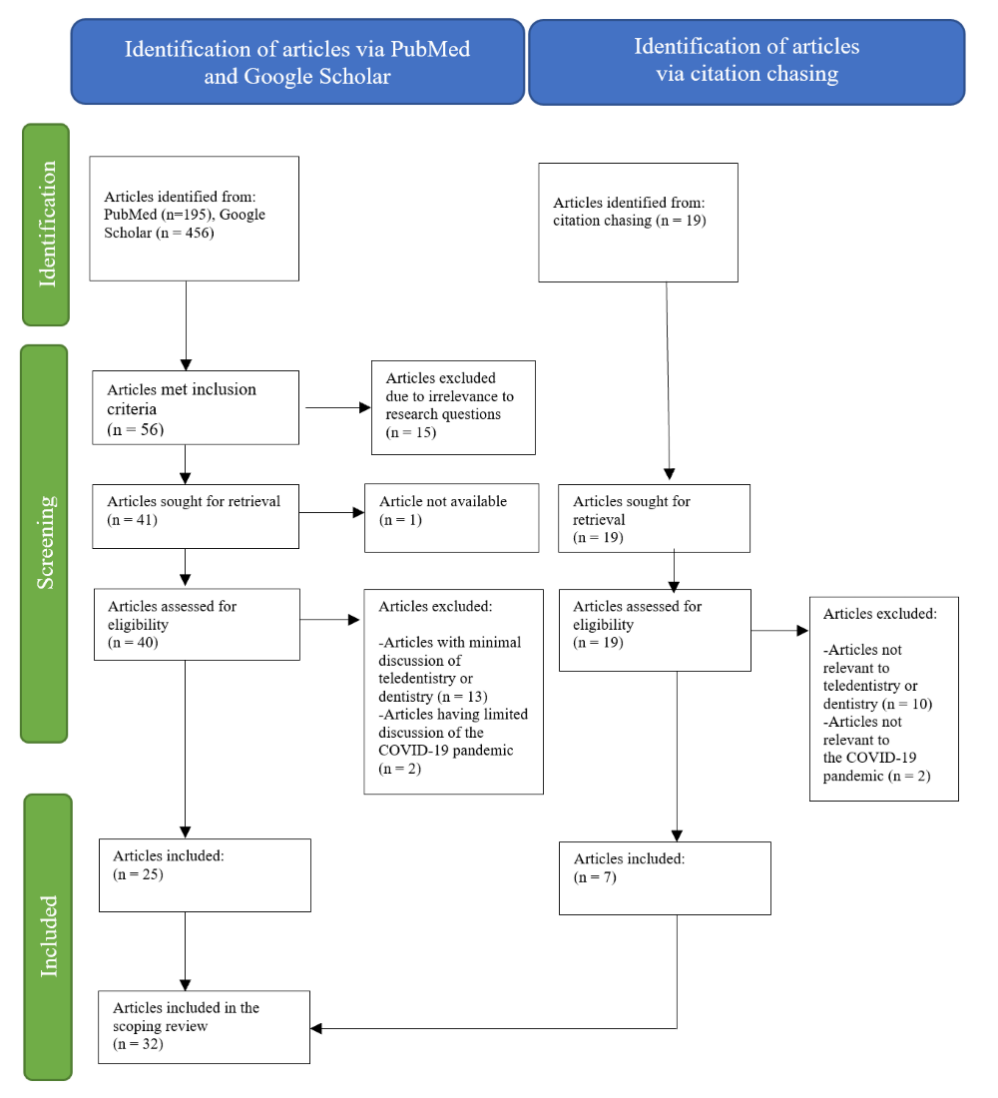
PRISMA flowchart for article selection. PRISMA: Preferred Reporting Items for Systematic Reviews and Meta-Analyses.

The flow chart in Figure 1 outlines the search strategy and reasons for excluding articles. Three reviewers (MN, TNP, and MSL) systematically appraised each article using a comprehensive form that included the full article citation, country, the type of article, a summary of key points, and, when applicable, the study population and research methodology. In addition, the authors used a checklist to ensure that each article contained key points related to one or more of the scoping review questions identified in the study aims. Following their independent review, the reviewers discussed any disagreements and achieved consensus. As part of this discussion, the authors noted that recurring key points emerged and noted the key points for each research question. For example, 3 recurring key points emerged for the question “How was teledentistry implemented during the COVID-19 pandemic?”: (1) modalities of teledentistry, (2) the applications and programs used to implement teledentistry, and (3) the reasons for using teledentistry. Articles addressing multiple study aims were reported for each aim they addressed.

Multimedia Appendix 2 details the 32 articles that met the selection criteria. Of the 32 articles, 10 were opinion pieces, 9 were narrative review articles, 4 were descriptive studies, 3 were surveys, 2 were integrative literature reviews, and there was 1 each of the following: observational study, systematic review, case report, and practice brief. The papers represented several countries including 8 from the United States, 5 from the United Kingdom, 4 from Italy and India, and 2 each from Brazil, the Kingdom of Saudi Arabia, and Canada. The remaining articles came from Pakistan, Iran, Hong Kong, Romania, and Malaysia.

### How Was Teledentistry Implemented During the COVID-19 Pandemic?

Many dental offices and institutions temporarily halted elective treatment to reduce COVID-19 spread and to preserve the supply of personal protective equipment. From the review, 3 groups of recurring items for teledentistry implementation emerged: (1) modalities such as synchronous and asynchronous, (2) the applications and programs used to implement teledentistry, and (3) the reasons for using teledentistry.

Live consultations (synchronous) or store-and-forward (asynchronous) use were discussed by 15 articles. Live consultations provided patients with the opportunity to interact with clinicians in real time, while in the store-and-forward option, patients provided photos, videos, texts, or voice messages that allowed clinicians to evaluate a patient’s concern at a convenient time and the ability to share images or radiographs with colleagues [5,16-25]. Radiographs used for teledentistry visits were prepandemic radiographs, and the inability to obtain new or additional images via virtual appointments was noted as a barrier to teledentistry [17,26]. If new or additional radiographs were required, patients scheduled an in-person visit for radiographic imaging [20,23].

The second recurrent item category was the type of applications and programs used to implement teledentistry. The most mentioned applications were WhatsApp, Skype, Zoom, and Google-related services [16,17,19,24,26-35]. Less common were Mobile Mouth Screening Anywhere, a program specifically designed to detect oral cancer via photos uploaded by patients; a video platform called Video 4 Health used in conjunction with a teletrailer program, Telegram; Attend Anywhere; Facetime; and Health Insurance Portability and Accountability Act (HIPAA)–compliant programs such as Mailgate SC, Doxy.me, WebEx, Virtru, MD Office Mail, and LuxSci [5,27,36,37]. Teledentistry for orthodontics used programs that tracked the progress of orthodontic treatment. Dental Monitoring, which applies artificial intelligence to monitor patients’ progress remotely, was used in Italy and the United States [22,29]. Other telecommunication platforms included GoToMeeting, BlueJeans, Microsoft Teams, and ReadyTalk [22]; Dentulu and Toothpick were used for scheduling orthodontic consultations [22]. Other applications mentioned included Smile Virtual, Review Tool, Smile Snap, and Rhinogram, all of which provide different features while allowing the clinicians to recruit patients asynchronously [22]. Teledentistry applications for patient management included TeleDent, Teledentix, and Carstack programs [22]. Features among these applications ranged from allowing clinicians to upload hygiene and health instructions, uploading documents for signatures, sending messages to patients, allowing patients to schedule appointments, and facilitating both synchronous and asynchronous consultations [22].

The third recurring item category was reasons for implementing teledentistry and included treatment, maintaining dental services, and triage. Two articles specifically focused on treating patients. In a pilot study based in Italy, Giudice et al [16] followed 2 groups of patients, one with urgent needs and a posttreatment group following a head and neck procedure, using teledentistry for initial consultations and WhatsApp to view pre- and postoperative radiographs and photographs. In Italy, Barca et al [31] used teledentistry for postoperative follow-up of precancerous lesions and for those with suspected oncological pathology or urgent head and neck concerns. These authors utilized WhatsApp or Telegram for text messaging and for transmitting images to assist in diagnosing, developing treatment plans, monitoring postoperative sites, and following suspicious lesions [31].

Other authors highlighted the integration of teledentistry into their existing systems to help maintain dental services during the COVID-19 pandemic. In the United Kingdom, Crawford and Taylor [5] described Attend Anywhere, a hospital-based teledentistry program, as a “virtual clinical system” supported by the National Health Service with YouTube tutorials to assist users. They provided video consultations for emergency clinics, new patient clinics, and multidisciplinary team clinics [5]. During a consultation, the dental professionals verified the patient’s identity, took a history, and, when needed, asked patients to email pictures to a secure account [5]. The dental professionals either managed the patient virtually or triaged the patient for an in-person appointment with an appropriate provider [5]. Gleeson and Kalsi [37] discussed Attend Anywhere’s ability to provide remote clinical consultation for patients with complex restorative needsf and patient satisfaction. In Brazil, physicians used telemedicine networks to provide coverage for all State municipalities. One network was Tele(oral)medicine [23], which had a goal of collecting data and communicating with dental professionals, who could make care recommendations and, for urgent issues, expedite referrals [23].

In India, the Armed Forces utilized teledentistry to triage patients [17], provide advice, and prescribe analgesics and antibiotics [17]. Patients contacted dental professionals using either the WhatsApp messenger application or email [17]. Dental professionals evaluated the patient’s concern and triaged the patient into either “Emergency Dental Treatment,” “Dependent Dental Centre,” or “Advice & Self Help” [17]. For example, if a patient reported difficulty swallowing or uncontrolled bleeding, the patient was referred for “Emergency Dental Treatment” [17], while those with less severe pain or dental trauma could be referred to a “Dependent Dental Centre.” Milder symptoms might be managed virtually through advice and self-help [17]. At the University of California San Francisco, a retrospective chart review found Tele(oral)medicine via Zoom helpful for pain management after an initial visit [26]. A United States study discussed integrating teledentistry into everyday private practice to mitigate pandemic-imposed restrictions using teledentistry both synchronously and asynchronously. The integration of teledentistry into the practice provided triage, some limited care, and hygiene assessments, as well as facilitating consultations with specialists [20]. Another pediatric and orthodontic practice separated dental professionals and staff members into 3 rotating teams [33] to answer calls and emails from patients. Patients could send photos and be followed up by phone, email, or video consultation using Zoom or FaceTime [33]. Researchers from Malaysia implemented Mobile Mouth Screening Anywhere, a web-based application that helps assess oral lesions. The app allows patients to upload photos of oral lesions and ask clinical questions; dental professionals review the data and provide recommendations [36].

Pediatric teledentistry applications and implementation were discussed by 3 articles [19,21,38]. Nuvvula and Mallineni [19] proposed using applications such as Facebook Messenger, Instagram, Skype, and WhatsApp to assess urgency and the appropriate response. One author in the United Kingdom used teledentistry to determine whether a patient required urgent or routine in-person care and if the case merited referral to other departments [21]. Parents could schedule appointments for their children, provide information regarding the child (pre- and postappointment), send images to a designated email address, and, if necessary, access BigWorld for interpretive services [21]. Kumar Mallineni et al [38] coined the term “telepediatric dentistry” for preventive services that gave parents advice and oral hygiene instructions to help maintain a child’s oral health.

Academic institutions, such as Oregon Health and Science University (OHSU), University of Washington (UW), and New York University (NYU) implemented teledentistry systems during the COVID-19 pandemic. OHSU and UW used HIPAA-compliant messaging and virtual consultation platforms and noted the importance of having appropriate audiovisual technology [35]. Staff members first triaged teledental patients, including COVID-19 screening for current symptoms, travel history, and recent exposures to those who tested positive [35]. Staff members followed an onboarding protocol using a HIPAA-compliant temporary account [35] and scheduled appointments during which patients logged in and received a teledental examination [35]. Dental professionals used a decision tree to guide next steps and follow-ups [35]. At NYU, patients could make an appointment for either a telephone or video consultation [39]. Teledentistry service agents triaged calls to the appropriate dental professionals or other specialist [39] who contacted patients either by phone or video call to discuss their concerns [39]. After hours or during weekends, patients left voicemails, and clinic personnel returned calls on the following day [39]. Findings for implementing teledentistry are summarized in Table 1.

**Table 1:** Article summary for scoping review research question 1 (implementation).

Key points	Article sources
Synchronous and asynchronous teledentistry implementation	Alsafwani et al (2022) [26] Chopra and Sahoo (2020) [17] Crawford and Taylor (2020) [5] Deshpande et al (2021) [18] Giudice et al (2020) [16] Meurer et al (2022) [23] Nuvvula and Mallineni (2021) [19] Park et al (2021) [22] Patel and Wong (2020) [24] Suter (2020) [20] Tonkaboni et al (2021) [25] Wallace et al (2021) [21]
Applications and programs used to implement teledentistry	Abbas et al (2020) [28] Alsafwani et al (2022) [26] Barca et al (2020) [31] Brecher et al (2021) [33] Caprioglio et al (2020) [32] Chopra and Sahoo (2020) [17] Chung et el (2022) [35] Crawford and Taylor (2020) [5] da Silva et al (2021) [30] Ghai (2020) [27] Giudice et al (2020) [16] Gleeson and Kalsi (2022) [37] Goriuc et al (2022) [34] Kumar Mallineni et al (2021) [38] Maspero et al (2020) [29] Nuvvula and Mallineni (2021) [19] Park et al (2021) [22] Patel and Wong (2020) [24] Rajendran et al (2022) [36] Torosyan et al (2021) [39] Wallace et al (2021) [21]
Reasons for implementing teledentistry	Alsafwani et al (2022) [26] Barca et al (2020) [31] Brecher et al (2021) [33] Chopra and Sahoo (2020) [17] Crawford and Taylor (2020) [5] Giudice et al (2020) [16] Gleeson and Kalsi (2022) [37] Meurer et al (2022) [23] Rajendran et al (2022) [36] Suter (2020) [20]

### What Challenges Occurred When Implementing Teledentistry During the COVID-19 Pandemic?

There were 4 major types of challenges: acceptance by dental professionals or staff, acceptance by patients, confidentiality, and reimbursement. The lack of acceptance of teledentistry by dental professionals or staff members was addressed by 8 articles. A common reason for not embracing teledentistry was a perceived difficulty in providing an accurate diagnosis based on videos or static images without being able to perform important diagnostic maneuvers such as percussion and palpation [18,19,24,27,30,31,40,41]. Additionally, images or videos may fail to fully visualize lesions and lesion borders or fail to capture 3-dimensionality and other pertinent information that might limit dental professionals’ ability to establish the correct diagnosis [16,18,24,30,34,36,41]. As a result, dental professionals were apprehensive about the potential for misdiagnosis, mismanagement, and litigation due to unfavorable treatment outcomes [21,24,29].

Other challenges included a lack of patient acceptance of teledentistry. Some patients may be unreceptive to video consultations due to an unfamiliarity with devices, and those with difficulty communicating, either because of language barriers or disabilities, may find it challenging to adapt to teledentistry [17,21,25,27,30,42,43]. Parents may be hesitant to use online consultations for their children because of unfamiliarity with teledentistry [19]. Teledentistry may also trigger anxiety and apprehension for proposed treatments and erode prepandemic patient-clinician relationships [19,21,24,30].

Protecting patient information such as photos, videos, or an electronic health history, was a common concern [5,6,18,24,25,29,30,42]. Many dental professionals utilized WhatsApp because of its end-to-end encryption. Park et al [22] noted WhatsApp, Apple FaceTime, and Facebook Messenger video chat are not HIPAA-compliant and may compromise patient information, while Zoom, Skype, and Google Meet offer HIPAA-compliant versions for dental professionals [22]. Moreover, for utilization of software that stores patient information, there should be a Business Associate Agreement (BAA) between the company providing the service and the dental professionals [22].

Several studies mentioned payment concerns and the need to discuss with insurance companies how to properly code virtual visits to ensure payment [5,18,27,41,42,44]. Most articles did not address how payment was made (whether it was by the patient or insurance companies) or how much the payment was but instead focused on the challenge of properly coding and billing for virtual visits. One article noted that teledentistry was often billed by using the code for the service provided such as D0140 (limited exam code) [7] and not teledentistry. However, this varied among different insurance carriers or states, and the authors advocated for more clarity regarding teledental visit reimbursement [7]. Additionally, many noted the cost of investing in the necessary technology infrastructure and the importance of having sufficient internet bandwidth to accommodate teledentistry transmission [5,6,17,19,21,24,27,30,34,35,41] since an inadequate internet connection may result in dropped service and a break in continuous treatment [5,6,17,28,30,31,34,41,44]. Lapses with the Attend Anywhere, which occurred during times of peak internet demand, was an example of dropped service [5]. Few guidelines for dispensing medications [17], a lack of appropriate scheduling software, and an insufficient number of trained staff were also identified as challenges [20,22]. Table 2 summarizes the challenges encountered in teledentistry.

**Table 2:** Article summary for scoping review research question 2 (challenges).

Key points	Article sources
Difficulties with acceptance by dental professional or staff	Barca et al (2020) [31] da Silva et al (2021) [30] Deshpande et al (2021) [18] Ghai (2020) [27] Giudice et al (2020) [16] Goriuc et al (2022) [34] Jajeh et al (2022) [40] Kumar et al (2022) [41] Maspero et al (2020) [29] Nuvvula and Mallineni (2021) [19] Patel and Wong (2020) [24] Rajendran et al (2022) [36] Wallace et al (2021) [21]
Difficulties with acceptance by patients	Chopra and Sahoo (2020) [17] da Silva et al (2021) [30] Ghai (2020) [27] Nuvvula and Mallineni (2021) [19] Patel and Wong (2020) [24] Samaranayake and Fakhruddin (2021) [43] Talla et al (2020) [42] Tonkaboni et al (2021) [25] Wallace et al (2021) [21]
Confidentiality	Crawford and Taylor (2020) [5] da Silva et al (2021) [30] Deshpande et al (2021) [18] Farooq et al (2020) [6] Maspero et al (2020) [29] Park et al (2021) [22] Patel and Wong (2020) [24] Talla et al (2020) [42] Tonkaboni et al (2021) [25]
Reimbursement	Abbas et al (2020) [28] Barca et al (2020) [31] Chopra and Sahoo (2020) [17] Chung et al (2022) [35] Crawford and Taylor (2020) [5] da Silva et al (2021) [30] Deshpande et al (2021) [18] Farooq et al (2020) [6] Ghai (2020) [27] Goriuc et al (2022) [34] Kumar et al (2022) [41] Nuvvula and Mallineni (2021) [19] Park et al (2021) [22] Patel and Wong (2020) [24] Singhal et al (2021) [44] Suter (2020) [20] Talla et al (2020) [42] Wallace et al (2021) [21] Wu et al (2020) [7]

### What Strategies Were Used to Overcome These Challenges?

Table 3 summarizes the 20 articles that discussed strategies to address challenges. These strategies included educating dental professionals, orienting patients, providing proper compensation, and updating technologies. To improve teledentistry acceptance, researchers suggested more education and dental professional training about teledentistry [27,36,42]. Kumar et al [41] proposed webinars and continuing education provided by the American Dental Association to improve dental professionals’ comfort with using newer technologies. Rajendran et al [36] required that dental professionals using Mobile Mouth Screening Anywhere review a refresher video about teleconsultation etiquette and regulatory requirements. Equally important may be training and education for patients and parents about how to transmit useful images and participate in video consultations [19]. To promote teledentistry, dental professionals and other specialists can spread awareness among patients about this option [36]. To alleviate apprehension, some suggested an introductory call or video tour to introduce the patient to the dental professionals and the environment or to allow patients more time to prepare for a consultation or a procedure [21,24,42]. For patients who speak a foreign language, translation services can create a more welcoming environment for patients [21,42]. To ensure dental professionals are properly compensated, authors from the United States and Canada shared specific codes that successfully resulted in compensation [33,41,44]. To prevent litigation, several authors emphasized the need to obtain informed consent that details the potential risks associated with online consultations [18,29,31,42]. Articles from the United States and Canada highlighted the importance of having a system in place to verify patient identity at the time of service to prevent disclosing sensitive information to someone other than the patient [35,41,42]. In a literature review, da Silva et al [30] recommended using Zoom accounts that require passwords to enter meetings and the ability to lock meetings once all parties are present. Several authors suggested using end-to-end encryption applications or devices, confirming that an application is HIPAA-compliant, and executing a BAA to maintain proper use of patient health information [16,22,29-31]. To improve diagnostic abilities, one author recommended that an iPhone 4S or later model is needed to make an adequate assessment [5]. However, dental professionals should be mindful that up-to-date technology incurs a financial burden on patients who may already be financially challenged by the pandemic [5]. Lapses in connection can occur, and a UK-based study found that they needed a more advanced server to manage virtual dental visits [5]. To address the lack of scheduling software, Suter et al [20] turned to project management software outside of dentistry. Strategies used to overcome challenges are summarized in Table 3.

**Table 3:** Article summary for scoping review research question 3 (strategies to overcome challenges).

Key points	Article sources
Educating dental professionals	Barca et al (2020) [31] Chung et al (2022) [35] Deshpande et al (2021) [18] Ghai (2020) [27] Kumar et al (2022) [41] Maspero et al (2020) [29] Rajendran et al (2022) [36] Talla et al (2020) [42]
Orienting patients	Nuvvula and Mallineni (2021) [19] Patel and Wong (2020) [24] Rajendran et al (2022) [36] Talla et al (2020) [42] Wallace et al (2021) [21]
Providing proper compensation	Brecher et al (2021) [33] Kumar et al (2022) [41] Singhal et al (2021) [44]
Updating technologies	Barca et al (2020) [31] Crawford and Taylor (2020) [5] da Silva et al (2021) [30] Giudice et al (2020) [16] Maspero et al (2020) [29] Park et al (2021) [22] Suter (2020) [20]

### Were There Innovative Ideas That Resulted From the Implementation of Teledentistry?

A major innovation was adapting commonly available programs and applications to teledentistry services. Applications such as WhatsApp or communication services such as Zoom were mentioned in several articles [16,17,19,24,27-32,35]. Other platforms mentioned included Telegram, Microsoft Network, Skype, and FaceTime [24,29,31,33]. These applications helped establish communication and played a role in reducing travel, especially in regions with travel restrictions or those that were difficult to access. With certain orthodontic emergencies, teledentistry provided a platform to advise patients about home remedies such as using a pencil eraser to push on metal ligatures causing soft tissue trauma or using sterilized nail clippers to remove protruding archwire [32]. As an ancillary benefit, telemedicine led to shorter wait times for pre- and postoperative evaluations and specialty consultations [16,18,19,28,29,31,33,37] and mitigated COVID-19 spread by reducing clinic overcrowding [17]. A Saudi Arabian clinic successfully used a questionnaire to triage patients and determine whether a pediatric patient needed in-person treatment [38]. They also provided previsit videos about COVID-19 protocols to protect all parties present in the dental office [38]. The implementation of virtual waiting areas was another teledentistry innovation [35,37,38]. For patients with concerns about oral pathology and who are uncomfortable with teledentistry, Goriuc et al [34] suggested home saliva testing to assess oncologic pathology.

Education is an important tool for maintaining oral health, and several authors suggested that teledentistry offers opportunities for education and community outreach to schools, nursing homes, rural residents, and those who are housebound as a result of age-related frailty or physical or mental disabilities [20,21,24]. Using teledentistry before a domiciliary visit can determine what materials dental professionals may need before making the visit [24]. Additionally, the synchronous and asynchronous capabilities of teledentistry helped dental institutions minimize pandemic-related lapses in dental education [6] and use virtual patients for dental students’ education [6]. Project management software outside the field of dentistry represented a possible solution to fill the gap in dental scheduling software [20]. The same article described virtual operatories and hybrid clinics that integrate in-person, synchronous, and asynchronous patient visits [20]. Teleorthodontic innovations included programs such as Dental Monitoring to scan a patient’s oral cavity and artificial intelligence to monitor treatment [22,29]. Others created their own programs. For example, Fazio et al [45] developed LinguAPP, which incorporated a patient questionnaire and the ability to upload photos to determine appropriate treatment.

Teledentistry was divided into subcategories such as teleconsultation, telediagnosis, teletriage, and telemonitoring services by 8 articles [17,19,21,22,27,28,30,43,44]. Through teleconsultation, dental professionals can address nonurgent concerns and use teletriage to prioritize patients needing urgent or emergency care so that they can be seen promptly [19,27,28,30,36]. Telediagnosis uses specific applications, such as Mobile Mouth Screening Anywhere, to help diagnose and share patient information, photos, videos, or radiographs with colleagues [19,27-29]. Telemonitoring can be utilized to follow patients who received advice or for follow-up of treatment [5,19,27,28,31]. However, the main role of teledentistry during the COVID-19 pandemic was to help bridge gaps in care, assure essential care for both new and existing patients, and broaden its scope to include dental specialties [17-20,28,30]. Teledentistry also expanded its use to include prescribing medications, such as antibiotics, pain medications, high-strength fluoride, and anti-inflammatory mouth rinses, which could temporarily address conditions such as swelling and pain until further treatment could be arranged [16-18,21]. Innovations are summarized in Table 4.

**Table 4:** Article summary for scoping review research question 4 (innovation).

Key points	Article sources
Adapting commonly available programs and applications to teledentistry	Abbas et al (2020) [28] Barca et al (2020) [31] Brecher et al (2021) [33] Caprioglio et al (2020) [32] Chopra and Sahoo (2020) [17] Chung et al (2022) [35] da Silva et al (2021) [30] Deshpande et al (2021) [18] Farooq et al (2020) [6] Ghai (2020) [27] Giudice et al (2020) [16] Gleeson and Kalsi (2022) [37] Goriuc et al (2022) [34] Kumar Mallineni et al (2021) [38] Maspero et al (2020) [29] Nuvvula and Mallineni (2021) [19] Patel and Wong (2020) [24] Suter (2020) [20]
Education and community outreach	Suter (2020) [20] Wallace et al (2021) [21] Patel and Wong (2020) [24]
Developing new teledentistry programs and applications	Park et al (2021) [22] Maspero et al (2020) [29] Fazio et al (2022) [45]
Separating teledentistry into subcategories of services (teleconsultation, telediagnosis, teletriage, and telemonitoring)	Crawford and Taylor (2020) [5] Chopra and Sahoo (2020) [17] Nuvvula and Mallineni (2021) [19] Suter (2020) [20] Wallace et al (2021) [21] Ghai (2020) [27] Abbas et al (2020) [28] Maspero et al (2020) [29] da Silva et al (2021) [30] Barca et al (2020) [31] Rajendran et al (2022) [36] Samaranayake and Fakhruddin (2021) [43] Singhal et al (2021) [44]
Expanding teledentistry to include prescribing medications	Giudice et al (2020) [16] Chopra and Sahoo (2020) [17] Deshpande et al (2021) [18] Wallace et al (2021) [21]

## Discussion

The purpose of this scoping review was to enhance the understanding of teledentistry use during the COVID-19 pandemic and examine challenges associated with expanded services and strategies to address these challenges. The COVID-19 pandemic also presented opportunities to enhance teledentistry’s scope, and this review explored innovative ideas that emerged from the expansion of teledentistry.

### Principal Findings

The results indicate that teledentistry offered important services to help maintain the continuity of dental care during the pandemic. Through the utilization of different applications, patients were able to communicate their concerns to dental professionals who could analyze photos, videos, and radiographs to triage those patients needing urgent or emergent care for an in-person visit. Several programs noted that teledentistry worked well for follow-up visits and, by reducing face-to-face visits, lowered the risk of viral transmission for patients, dentists, and office staff [5,16-25].

One unexpected benefit was improved access to specialists through teledentistry triage, enhanced communication, and the use of asynchronous data transmission. In addition to mitigating the pandemic’s impact on staff exposure and reducing in-person visits, teledentistry decongested clinics, which may be useful for improving patient flow postpandemic. Another benefit of teledentistry was its ability to reduce travel and the financial burdens associated with travel and improve access for patients with disabilities or other barriers to in-person visits [16,18,19,29]. Teledentistry was identified as effectively addressing concerns such as identifying clearly benign growths and providing reassurance, removing the need for an in-person visit. Although articles on teledentistry implementation reported favorably on its role in bridging gaps in care caused by the pandemic, its implementation elicited several concerns. Acceptance by both dental professionals and patients may prove to be an obstacle that hinders future expansion, and security concerns about patient health information may also impede the acceptance of teledentistry [16,18,19,24,27,30,31].

A consistent and clear barrier to teledentistry implementation was the inability to perform diagnostic tests such as percussion, palpation, and thermal testing. The variation in image and video quality and the potential for misdiagnosis, mismanagement, and litigation were also identified as issues that may jeopardize widespread acceptance [21,24,29]. These results highlight the need for guidelines and an accepted scope of services that make teledentistry a safe and reliable platform for both dental professionals and patients. Although this review identified roles for triage, diagnosis, monitoring, and prevention, examples of conditions that might be included in a defined scope of teledentistry services include temporomandibular joints, pain management, and prescribing antibiotics. The need for advocacy with governing dental regulatory bodies is important to support and endorse protocols for teledentistry, including guidelines for dispensing medications and treatment [17,42]. Clear guidelines and protocols recommended by governing dental bodies for treatment, prescriptions, and dental codes can facilitate the process for those interested in implementing teledentistry and continuing programs beyond the pandemic. Having dental regulatory agencies certify applications and platforms for dental services as HIPAA-compliant may accelerate teledentistry acceptance by dental professionals [5]. Despite the recent adoption of dental procedure codes for teledentistry for both synchronous and asynchronous delivery of dental services, reimbursement remained a commonly mentioned concern. Codes alone may not be sufficient, since Singhal et al [44] mentioned reimbursement as an issue in Canada even though the Canadian Dental Association published insurance codes for teledentistry. In the United States, some states including California endorsed Medicaid reimbursement for teledentistry services, and although this likely helped reimbursement, no paper evaluated the impact of these legislative acts during the study period.

One outcome from the enhanced use of teledentistry during the pandemic is that it will likely contribute to an enduring and broader role in the future. In 2020, about one-third of United States adults used teledentistry, and over 80% of patients reported being satisfied with their teledental visit and indicated a willingness to use the modality again [46]. Almost three-quarters of dentists who used teledentistry anticipated maintaining or even increasing their virtual visits [47]. However, many emergency regulations, such as relaxing licensing requirements and HIPAA compliance, will be expiring, and there is debate on how to proceed moving forward. This review supports the value of teledentistry and suggests that further development of this modality can contribute to improved oral health postpandemic.

### Limitations

A limitation of this scoping review was the exclusion of articles outside of PubMed and Google Scholar. Though the review included opinions and letters to the editor, it excluded government reports, conference proceedings, and policy statements. However, “grey” literature does not undergo the same rigorous peer review as more traditional academic literature. Selection bias of articles on oral surgery may also be present since several retrieved articles were identified by the search term of telemedicine. These articles met the inclusion criteria because they incorporated the oral maxillofacial surgery specialty but no other dental specialties. Finally, this review focused on teledentistry and its implementation and uses during the lockdown period. By excluding articles that did not incorporate teledentistry and its connection to COVID-19, extrapolating the results beyond the pandemic or applying the finding to future pandemics is speculative.

### Conclusions

The COVID-19 pandemic lockdowns led to new teledentistry implementations, most commonly for triage but also for follow-up and nonprocedural care. Teledentistry improved access to dental services and reduced in-person visits. However, significant challenges remain. These challenges include insufficient technology infrastructure, inadequate provider skill, and a lack of established protocols to manage billing issues and privacy concerns. As pandemic restrictions ease, these barriers will need to be addressed to sustain and expand teledentistry.

## Appendix

Multimedia Appendix 1 PRISMA checklist for scoping review. PRISMA: Preferred Reporting Items for Systematic Reviews and Meta-Analyses.

Multimedia Appendix 2 Article summaries.

## Abbreviations

BAA: Business Associate Agreement
HIPAA: Health Insurance Portability and Accountability Act
NYU: New York University
OHSU: Oregon Health and Science University
PRISMA-ScR: Preferred Reporting Items for Systematic reviews and Meta-Analyses for Scoping Reviews
UW: University of Washington
