# Supplementary material for: Teledentistry Implementation During the COVID-19 Pandemic: Scoping Review
Source: Interact J Med Res. 2022 Jul 21;11(2):e39955. doi: 10.2196/39955 (PMC9307266; doi:10.2196/39955)
Supplement: Multimedia Appendix 2 [file ijmr_v11i2e39955_app2.docx]

| Article Number | Author (Year) | Title | Article Type | Methodology and Population | Outcome of Scoping Review Questions^a^ |
| --- | --- | --- | --- | --- | --- |
| 1 | Abbas B. et al. (2020) | Role of Teledentistry in COVID-19 Pandemic: A Nationwide Comparative Analysis among Dental professionals | Survey | A prevalidated structured questionnaire was administered to 510 dental professionals | 2. Patient privacy and security, appropriate internet infrastructure, and adequate training required for the implementation of teledentistry  4. Incorporation of WhatsApp for teledentistry |
| 2 | Alsafwani Z. et al. (2022) | The role of telemedicine for symptoms management in oral medicine: a retrospective observational study | Retrospective observational study | Retrospective chart review of 137 new patients | 1.  Tele(oral)medicne is effective for symptom management of oral medicine conditions |
| 3 | Barca I. et al. (2020) | Telemedicine in Oral and Maxillo-Facial Surgery: An Effective Alternative in Post COVID-19 Pandemic | Survey | 90 Maxillofacial surgery outpatients divided into two groups: those who required follow-ups and those with suspected pathology | 1.Good satisfaction for both patients and doctors  2.Cannot do a complete clinical evaluation and internet connection issues  3.Improved documentation and improved software helped overcome inability to complete clinical evaluation.  4.Encrypted applications (e.g., WhatsApp) increase security |
| 4 | Brecher E.A. et al. (2021) | Teledentistry Implementation in a Private Pediatric Dental Practice During the COVID-19 Pandemic | Descriptive Study | Documented the management and modality of care for 137 emergency visits in a private pediatric practice during COVID-19 | 1. Nearly half of the emergency encounters were managed with teledentistry |
| 5 | Capriolglio A. et al. (2020) | Management of orthodontic emergencies during 2019-NCOV | Opinion | Summarized guidelines - WHO and Italy | 1. WhatsApp good method for triage and emergency management |
| 6 | Chopra S.S. et al. (2020) | Protocol for Teledentistry during COVID-19 in Armed Forces dental establishments | Opinion | Armed service members in India | 1.Used for triage. Helps mitigate lockdown limitations by providing communication, improved scheduling  2. Lack of direct contact between patients and clinicians, adequate internet connection needed, deficient patient knowledge of technology, inability to obtain diagnostic radiographs, and few prescription guidelines  4. WhatsApp, emails, phone calls as virtual services. Staggered clinic schedule to prevent overcrowding |
| 7 | Chung K. et al. (2022) | Teledentistry: An Adjunct for Delivering Dental Care in Crises and Beyond | Opinion | Clinics Oregon Health and Science University and the University of Washington | 1. Outlined process. The front desk triages, schedules appointments, sets expectations. The clinician does history and uses show, tell, and exam format. Refers as needed for in-person exam. System worked.  2.Billing issues, provider training, patients need high-speed internet, difficulty for patients to log in to virtual appointments  4.Can reduce costs and expand access |
| 8 | Crawford E. et al. (2020) | The effective use of an e-dentistry service during the COVID-19 crisis | Descriptive study | Retrospective chart review of 300 UK dental patients by two clinicians | 1. Established a digital workflow using Attend Anywhere. Successful emergency clinics led to new patient treatment reviews and multidisciplinary team clinics.  2. Specific hardware required for teledentistry (i.e., PC’s preferred), internet connection issues. Orthodontic services limited since patient interaction is required for appliance adjustment and most orthodontic care.  3. Connection hosts changed to alleviate internet issues |
| 9 | Da Silva H.E.C. et al. (2021) | The role of teledentistry in oral cancer patients during the COVID-19 pandemic: an integrative literature review | Integrative Literature Review | Searched articles on PubMed, Cochrane, Scopus, Web of Science, Lilacs, Embase, Open Grey, Google Scholar, and Jstor databases | 2. Poor internet connection, lack of training, issues with insurance reimbursement, limitations to patient evaluation, patients’ apprehension of teledentistry, and inability to perform key diagnostic tests (palpation and percussion)  3. Encryption of photos/data exchange and limited number of people accessing data  4. Used corporate version of Zoom for privacy, held multidisciplinary meetings, and allowed prioritization of patients in need of in-person treatment |
| 10 | Deshpende S. et al. (2021) | Teledentistry: A boon Admidst COVID-19 Lockdown – A Narrative Review | Narrative Review | Described the importance of teledentistry for general and  specialized dentists to manage dental  emergencies during lockdown | 1. Aids in emergency management, facilitates specialty consultation, reduces follow-up visits  2. Accuracy of virtual examinations, inability to perform percussion and palpation, noted litigation and confidentiality issues concerns,  Helps mainly for prevention and diagnosis. Clinic visits needed for procedural treatment3. Obtain informed consent  4. Prescription of antibiotics and home care instructions, increased access to specialists, virtual follow-up appointments |
| 11 | Farooq I. et al. (2020) | COVID-19 outbreak, disruption of dental education, and the role of Teledentistry | Narrative Review | Summarizes the potential role of teledentistry  in dental education | 1. Reduced patient care costs, increased access in rural areas, decreased disruption in dental education  2. Poor internet connectivity, need to train instructors in modality, limitations to clinical training of students, security of patient information  4. Deliver asynchronous and synchronous lesson plans, collect patient data as learning material, and helps train students to interact with patients |
| 12 | Fazio M. et al. (2022) | LinguAPP: An m-Health Application for Teledentistry Diagnostics | Descriptive Study | Development of a mobile application | 4. Development of LinguApp to gather diagnostic information and triage patients |
| 13 | Ghai S. (2020) | Teledentistry during COVID-19 Pandemic | Narrative Review | Literature search from PubMed, Google Scholar, and Cochrane database. Provides a brief overview of teledentistry during the pandemic | 2. Lack of acceptance by dentists and patients, inadequate infrastructure, insurance reimbursement, implementation costs, inability to perform percussion and palpation  3. Dentists need training and incorporation of teledentistry into dental school education  4. Use of applications such as Mobile Mouth Screening Anywhere, WhatsApp |
| 14 | Giudice A. et al. (2020) | Can Teledentistry Improve the Monitoring of Patients during the Covid-19 Dissemination? A Descriptive Pilot Study | Descriptive Study | Descriptive study, two population groups: adult patients with urgent problems and surgical follow-up | 1. Evaluated urgent problems, monitored patients, and post-surgical follow-up.  2. Images may be inadequate, medicolegal concerns  3. WhatsApp end-to-end encryption, limit access to mobile devices with patient data, data transferred to secure hard drive  4. Use of WhatsAPP, electronic prescribing |
| 15 | Gleeson H.B. et al. (2022) | Remote clinical consultations in restorative dentistry | Survey | Surveyed patients treated for head and neck cancer and cleft lip and palate | 1. Remote consultations were highly valued by patients. High patient acceptability. |
| 16 | Goriuc A. et al. (2022) | The Impact of the COVID-19 Pandemic on Dentistry and Dental Education: A Narrative Review | Narrative Review | Summarizes COVID-19’s impact  with an emphasis on  protocols, technologies, and their future implications on patient care | 1. Enhanced access to patients with access barriers  2. Lack of guidelines, privacy concerns, inability to perform tactile diagnostic tests, misdiagnosis, infrastructure and internet concerns  3. When remote consultations cannot be performed, conduct saliva tests to assist in determining oral pathology  4. Olohealth useful new platform, expanded e-learning |
| 17 | Jajeh N. et al. (2022) | Oral cancer diagnosis amid COVID-19 pandemic: Identifying tell-tale signs to avoid pitfalls in general dental practice | Case Report | 74-year-old male post-wisdom extraction | 1. Without face-to-face  consultations a video  consultation helps evaluate a complaint  2. Proper diagnosis of pathology. Suspected cancer needs in-person evaluation |
| 18 | Kumar Mallineni S. et al. (2020) | Dentistry for children during and post COVID-19 pandemic outbreak | Narrative Review | Reported in pediatric dentistry during the pandemic including teledentistry | 1. Used teledentistry questionnaire  4. Screen patients and guardians before face-to-face visits, “digital arena” or digital waiting area to notify patients when to enter the clinic |
| 19 | Kumar P. et al. (2022) | Teledentistry at the Crossroads: Benefits, Barriers, and Beginnings | Opinion | Discussed usage and implementation of teledentistry | 1. Useful for triage, monitoring, and identifying abnormal lesions  2. Internet speeds, technological literacy, privacy concerns, the accuracy of using online modalities  3. ADA resources (webinars to increase clinician comfort with using teledentistry), continuing education courses, should develop a way to confirm the identity of patients for security reasons  4. pandemic “silver lining” for teledentistry |
| 20 | Maspero C. et al. (2020) | Available Technologies, Applications, and Benefits of Teleorthodontics. A Literature Review and Possible Applications during the COVID-19 Pandemic | Systematic Review | Literature search using Medline, Pubmed, Embase, Cochrane Library, EBM Reviews, Web of Science, Ovid, and Google Scholar to the efficacy of teleassistance in orthodontics | 2. Medico-legal implications and patient confidentiality  3. Antivirus firewalls or applications with encryption  4. Dental Monitoring, an application with artificial intelligence to monitor patient treatment progress, use of MSN, Skype, and Whatsapp |
| 21 | Meurer M.I. et al. (2020) | Launching public statewide tele(oral) medicine service in Brazil during the COVID-19 pandemic | Opinion | Described teledentistry model in Brazil | 1. Identifying and prioritizing patients with serious conditions |
| 22 | Nuvvula S. et al. (2021) | Remote management of dental problems in children during and post the COVID-19 pandemic outbreak: A teledentistry approach | Narrative Review | Literature search with PubMed | 2. Acceptance by pediatric patients, parents, dentists, improper diagnosis, technical issues, implementation costs  3. Technical training for a pediatric dentist and team and parents in proper pictures and video-calling  4. Increased access to pediatric specialists in remote areas |
| 23 | Park J.H. et al. (2021) | Teledentistry Platforms for Orthodontics | Narrative Review | Overview for orthodontists considering remote practice | 2. asynchronous capabilities are lacking, not useful for long term care, inability to sync certain applications with current practice’s management software, HIPAA concerns  3. Make guidelines for teledentistry use, HIPAA compliant applications  4. Dental Monitoring, various platforms for orthodontics (zoom, ToothPic, Smile Virtual, Teledent, etc.) |
| 24 | Patel T. et al. (2020) | The role of real-time interactive video consultations in dental practice during the recovery and restoration phase of the COVID-19 outbreak | Opinion | Discussed interactive video consultations during the recovery and restoration phase of the COVID-19 outbreak | 2. Proper diagnosis, lack of clinician acceptance, fear of lawsuits, and financial reimbursements  3. Useful to introduce anxious patients to the dental setting, determine what treatment needs to be completed before a domiciliary visit  4. Reduce dental anxiety, document what instruments are needed before a domiciliary visit, use of Skype, WhatsApp, and FaceTime |
| 25 | Rajendran S. et al. (2022) | Accelerated implementation of teleconsultation services for the monitoring of oral potentially malignant disorders as a result of the COVID-19 pandemic | Opinion | Assessed patients attending oral medicine and surgery clinic in Malaysia | 1. Providing options for patient care, patient engagement, and empowerment.  2. Accuracy of diagnosis and photos, and acceptance of teleconsultations  3. Courses on online etiquette and Mobile Mouth Screening Anywhere application  4. Brochures with information about online consultations |
| 26 | Samaranayake L. et al. (2021) | Pandemics past, present, and future | Narrative Review | Reviewed literature about pandemics | 2. Reluctance of patients and providers |
| 27 | Singhal S. et al. (2021) | Reviewing Teledentistry Usage in Canada during COVID-19 to Determine Possible Future Opportunities | Integrative literature review | Environmental scan across all Canadian jurisdictions | 2. Limits of teleconsultations, increased costs, privacy and security concerns, internet connectivity, and reimbursement  3. Certain Canadian jurisdictions developed guidelines for implementation, Canadian Dental Association published accurate codes for insurance |
| 28 | Suter N. (2020) | Teledentistry applications for mitigating risk and balancing the clinical schedule | Practice Brief | Description of a clinic utilizing teledentistry | 1. Helped triage patients, consults, outreach, specialist consultations  2. Gaps in dental software to manage teledentistry  4. Project management tools from other industries incorporated into a dental practice, created “virtual operatories” for synchronous consultations |
| 29 | Talla P.K. et al. (2020) | Delivering dental care as we emerge from the initial phase of the COVID-19 pandemic: Teledentistry and face-to-face consultations in a new clinical world | Opinion | Discussed future directions for crises such as the COVID-19 and future implementation of teledentistry beyond the pandemic | 2. Clinician and patient acceptance, patient privacy, insurance recognition of teledentistry  3. Further research needed, training/continuing education courses, temporary insurance codes put in place by dental bodies, protocols to ensure correct consultations and patients are being seen |
| 30 | Tonkaboni A. et al. (2021) | Teledentistry: during COVID-19 | Opinion | Commented on value | 1. Reduce unnecessary in-person visits, reach patients in remote areas, and accurate triage |
| 31 | Torosyan S. et al. (2021) | The Challenges and Rewards of Teledentistry | Opinion | Discussed experiences at NYU and noted challenges | 1. Helps with emergency care during weekends/office closures. Triage patients  2. Implementation costs, insurance reimbursement, relationship/trust between patients and clinicians, patient privacy  3. Research state laws on teledentistry, review insurance reimbursement requirements, proper training of the dentist, security measures for remote working |
| 32 | Wallace C.K. et al. (2021) | Role of teledentistry in paediatric dentistry | Narrative Review | Service evaluation of new patient teledentistry appointments | 1. Successful contact with patients, appropriate appointments scheduled. Reduced unnecessary in-person visits  2. Misdiagnosis, mismanagement of patient, legal issues  3. Diagnosis and management of patients only when applicable  4. Delivery of preventive advice, reduce patient anxiety, allow non-English speakers to have an interpreter |

^a^Scoping Review Questions addressed: 1= implementation; 2= challenges; 3= strategies to overcome challenges; 4= innovation.
